# Diatom diversity through HTS-metabarcoding in coastal European seas

**DOI:** 10.1038/s41598-018-36345-9

**Published:** 2018-12-24

**Authors:** Roberta Piredda, Jean-Michel Claverie, Johan Decelle, Colomban de Vargas, Micah Dunthorn, Bente Edvardsen, Wenche Eikrem, Dominik Forster, Wiebe H. C. F. Kooistra, Ramiro Logares, Ramon Massana, Marina Montresor, Fabrice Not, Hiroyuki Ogata, Jan Pawlowski, Sarah Romac, Diana Sarno, Thorsten Stoeck, Adriana Zingone

**Affiliations:** 1 Stazione Zoologica Anton Dohrn, Villa Comunale, 80121 Naples, Italy; 2 CNRS, Aix-Marseille Université, IGS UMR7256 (IMM FR3479), F-13288 Marseille, France; 3 Cell & Plant Physiology laboratory, UMR 5168, University of Grenoble Alpes, CNRS, CEA, INRA, Cedex 9, 38054 Grenoble, France; 4 Sorbonne Université, CNRS, Station Biologique de Roscoff, AD2M, UMR 7144, Place Georges Teissier, 29680 Roscoff, France; 5 Department of Ecology, Kaiserslautern University of Technology, Erwin-Schrodinger Str. 14, D-67663 Kaiserslautern, Germany; 6 Department of Biosciences, University of Oslo, P.O. box 1066 Blindern, N-0316 Oslo, Norway; 7 Institut de Ciències del Mar (CSIC), Passeig Marıtim de la Barceloneta, 37–49, ES-08003 Barcelona, Catalonia Spain; 8 Institute for Chemical Research, Kyoto University, Uji, 611-0011 Japan; 9 Department of Genetics and Evolution, University of Geneva, 4, Boulevard d’Yvoy, CH-1211 Geneva, Switzerland

## Abstract

Diatoms constitute a diverse lineage of unicellular organisms abundant and ecologically important in aquatic ecosystems. Compared to other protists, their biology and taxonomy are well-studied, offering the opportunity to combine traditional approaches and new technologies. We examined a dataset of diatom 18S rRNA- and rDNA- (V4 region) reads from different plankton size-fractions and sediments from six European coastal marine sites, with the aim of identifying peculiarities and commonalities with respect to the whole protistan community. Almost all metabarcodes (99.6%) were assigned to known genera (121) and species (236), the most abundant of which were those already known from classic studies and coincided with those seen in light microscopy. rDNA and rRNA showed comparable patterns for the dominant taxa, but rRNA revealed a much higher diversity particularly in the sediment communities. Peculiar to diatoms is a tight bentho-pelagic coupling, with many benthic or planktonic species colonizing both water column and sediments and the dominance of planktonic species in both habitats. Overall metabarcoding results reflected the marked specificity of diatoms compared to other protistan groups in terms of morphological and ecological characteristics, at the same time confirming their great potential in the description of protist communities.

## Introduction

Environmental rDNA metabarcoding has opened an entirely new way of assessing microbial diversity in natural environments. The method provides data on many organisms that so far escaped our attention because they are difficult to identify with classic methods, hard to culture, fragile, or rare^1,2^. For many protistan lineages, metabarcoding is actually the first method that revealed their distribution in the world’s oceans (e.g., Diplonemida^3^ and Collodaria^4^). A quite different scenario is presented by diatoms, which were among the first microbes detected in the sea and by far the most deeply investigated^5,6^. The rigid siliceous frustule allowed these organisms a much higher level of morphological diversity in comparison with other protistan groups. Differences in shape, presence of appendages and fine ornamentation, observable in light and electron microscopy, were used as the base of a well-established taxonomy; the ease with which they can be grown and studied in culture greatly aided their molecular identification and the reconstruction of their phylogenetic relationships^7–9^. In addition, diatom distribution in coastal and open marine waters was the object of numerous studies, stimulated by their abundance and extensive distribution patterns, as well as their important contribution to the primary production and biogeochemical cycles in these habitats.

Metabarcoding is offering new insights in diatom diversity as well, shedding light on distribution and biogeographic patterns of species difficult to identify with optical methods^10,11^ and extending the exploration of their diversity over much larger and so far scarcely investigated oceanic areas^12^. High throughput sequencing (HTS) of rDNA barcodes is producing a large number of sequence tags that can be used for diatom identification at the genus or even the species level, depending on the marker, thus allowing for an unprecedented detailed appraisal of the species present in a sample or an area, compared with optical methods. At the same time, the objective identification offered by the molecular signature reflects the actual diversity of diatom species far more precisely. Finally, the use of datasets collected and analysed with the same methodology across different geographic areas allows diversity assessment over wide regions and makes comparisons sounder^13–15^.

In this study we analysed diatom distribution and diversity around European coasts using a complex environmental metabarcode dataset collected within the EU BIODIVERSA project BioMarKs. Barcodes of the hyper-variable V4 region of the 18S rRNA locus were obtained from samples from different size fractions, water column depths and sediments on eight dates at six European coastal sites in the Mediterranean (Blanes and Naples), Atlantic (Gijón and Roscoff), Skagerrak (Oslo) and Black Sea (Varna). Previous studies on the same dataset analysed the overall composition of coastal protist communities^16^, the pattern of distribution of rare species^1^, the diversity of protists in the sediments^17^ as well as individual protistan groups or species (e.g.^10,18^,). In the present study, we focused on diatoms and addressed a number of questions: What is the overall composition and geographic distribution of diatom communities at the sampling sites? Are there differences between the performances of rDNA and rRNA templates? What diatom species are present in the different size fractions? Which is the relationship between diatom communities in the water column and sediments? The general aim of this effort was to attempt a first definition of the coastal European diatom communities and identify the specific biological features of diatoms emerging from HTS-metabarcoding analyses in comparison with those recognized for the whole protistan community analysed so far.

## Results

The diatom V4 dataset used for the analyses consisted of 89 samples collected at the six sampling sites, two of which, Oslo and Naples, were sampled in two different seasons. Water column samples (subsurface and DCM) with the respective size fractions (pico, nano and micro-meso plankton) consisted of 73 samples (37 rDNA and 36 rRNA). Sediment samples consisted of eight rDNA and eight rRNA samples. The whole dataset contained 143,036 total diatom reads (25,606 distinct metabarcodes, or unique ribotypes) split in 53,878 rDNA and 89,158 rRNA reads. Details of samples and diatom reads used in our analyses are shown in Supplementary Tables S1–S3.

Overall, diatoms in coastal European waters accounted for about 19% of total protist reads, with their percentage varying among sites and seasons between 0.24% (Gijón, September 2010) and 42.5% (Naples, October 2009) (Supplementary Fig. S1). Reads from Naples and Oslo, the two sites sampled twice, represented 75% of the total diatom dataset. The proportion of diatom reads was higher in the sediments (40%) than in the water column (15%).

### Taxonomic analyses

The taxonomic assignment of ribotypes revealed a total of 121 genera (all habitats, size fractions and templates). The majority of ribotypes (82%, corresponding to 91% of the reads) were assigned to reference sequences at similarity level between 98 and 100%, producing a list of 236 species. The remaining ribotypes were assigned to known genera and in 4 cases ribotypes could be assigned only as “araphid pennate” and in 2 cases only to “uncultured diatom”. The final assignment produced a list of 340 different taxa (Supplementary Table S4).

**Figure 1:**
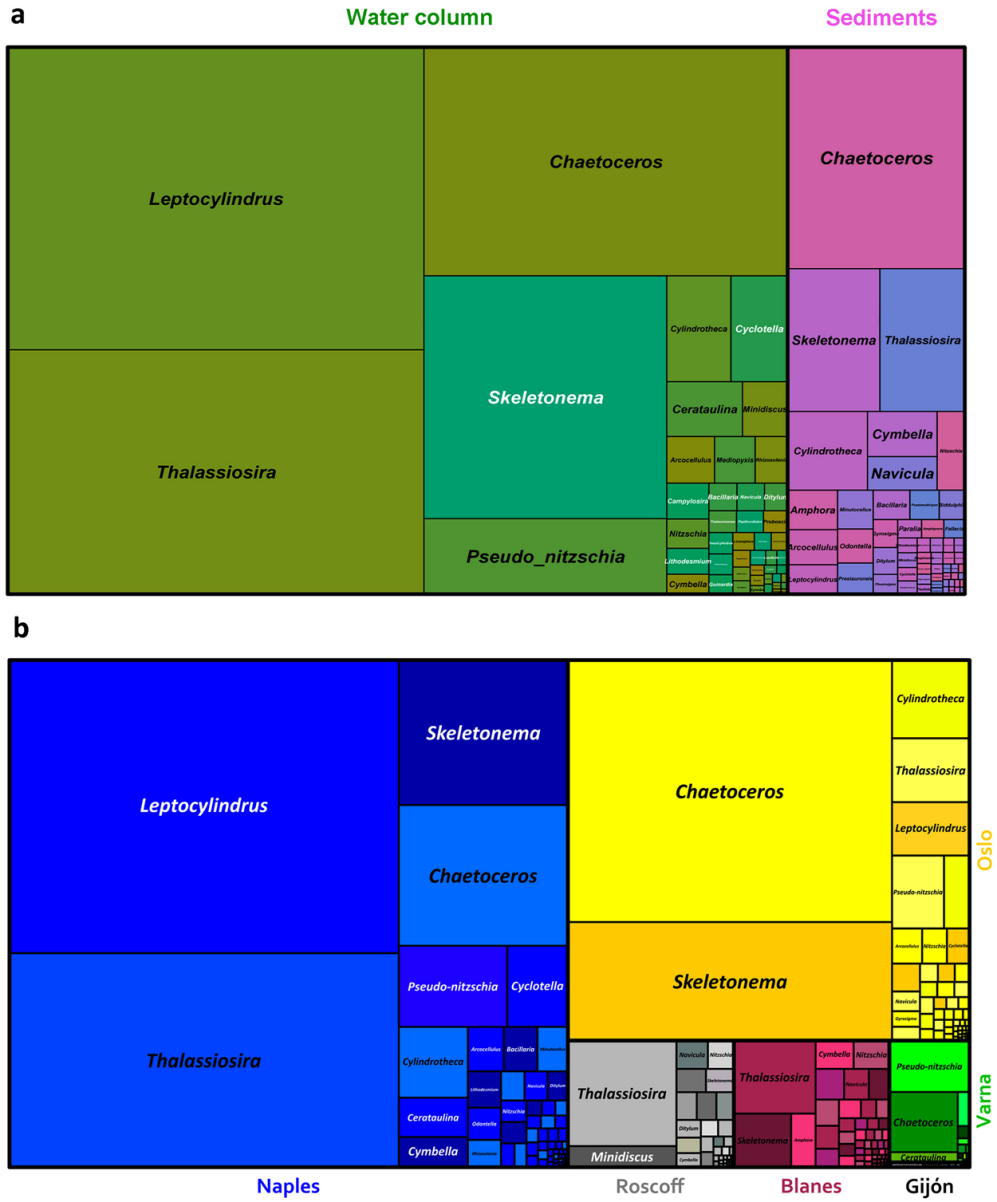
Distribution of diatom genera in coastal European seas. (**a**) Distribution of genera in the whole water column (green nuances) and sediment (pink nuances) datasets, both dominated by planktonic genera (*Leptocylindrus*, *Chaetoceros*, *Thalassiosira* and *Skeletonema*). (**b**) Different contribution of diatoms and relative abundance of main genera (including water column and sediments) at European sampling sites (Naples = blue, Oslo = yellow, Roscoff = grey, Blanes = purple, Varna = green, Gijon = black). Naples and Oslo samples collected in two different seasons were merged.

**Figure 2:**
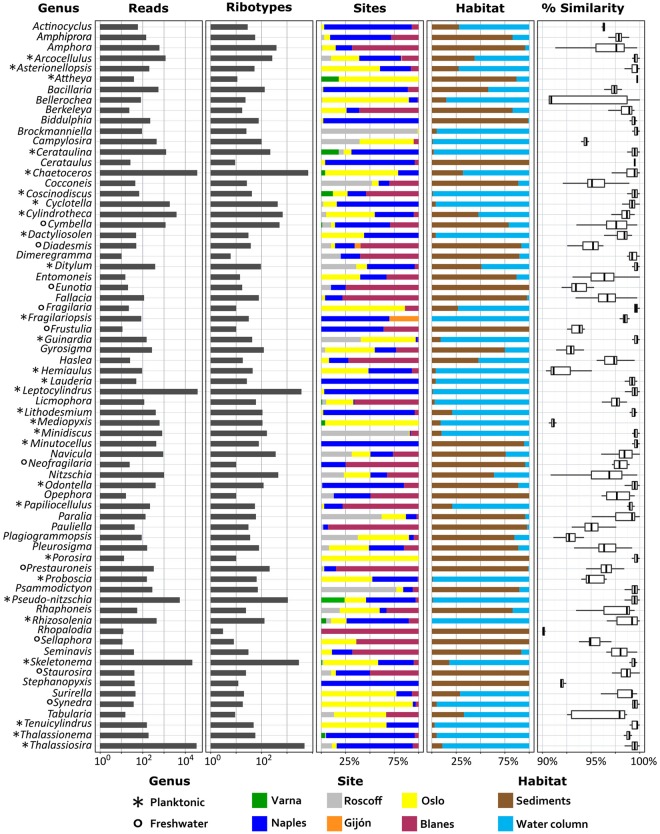
Abundance and distribution of the main diatom genera at coastal European sites. All ribotypes (summing all habitats, size fractions and templates) are clustered at genus level and only the 70 genera represented by more than 9 reads are shown. The colour scheme for the sites reflects the one of Fig. 1b. The rightmost column shows the similarity of the reads to known reference sequences of the genera.

The most abundant genera in the whole dataset were *Leptocylindrus*, *Chaetoceros* and *Thalassiosira*, which represented 23.8, 24.9 and 19.9% of the total diatom reads, respectively, followed by *Skeletonema* (14.6%) and *Pseudo-nitzschia* (3.7%) (Figs 1, 2). The next 65 genera represented 13.6% of total assigned reads, though 51 genera corresponded to less than 10 reads each. This overall picture reflected the distribution of diatoms in water column samples, which included 80% of the diatom reads (Fig. 1a). The sediment communities were still dominated by planktonic taxa, with *Chaetoceros* as by far the most abundant genus (40%), followed by *Thalassiosira* and *Skeletonema* representing 12.4% and 13.4% of the total diatom reads, respectively. *Leptocylindrus* and *Pseudo-nitzschia* were instead barely detected (1.50 and 0.15%, respectively). The remaining 32.6% of reads from sediments were assigned to a large number of genera (100), many of which are known to inhabit the benthic environment (e.g., *Nitzschia*, *Navicula* and *Amphora*). A number of reads (0.27% of the water column and 4.89% of the sediment reads) were assigned to freshwater genera, in most cases with low similarity with the reference sequence (Fig. 2). The average similarity to reference sequences for benthic genera was much lower than that for planktonic genera.

Diatom community composition showed different patterns at individual sites (Figs 1b and 2). *Leptocylindrus* dominated the diatom community at the Naples and Gijón sites, *Chaetoceros* at Oslo and Varna, *Pseudo-nitzschia* at Varna; *Thalassiosira* dominated at the Roscoff site but was also abundant at Blanes and Naples. A quite long list of taxa was identified through metabarcoding (Supplementary Table S4), including species not known so far at individual sites. Among these were *Papiliocellulus simplex* and *Thalassiosira concaviuscula* in Naples, several *Skeletonema* and *Chaetoceros* species in Oslo and many benthic taxa at both sites. Taxonomic assignment at species level was at times ambiguous because the V4 region may be shared between species, as for example in the case of *Skeletonema tropicum* and *S*. *pseudocostatum*, or *Pseudo-nitzschia multiseries* and *P*. *australis* (Supplementary Data Files S1 and S2).

**Figure 3:**
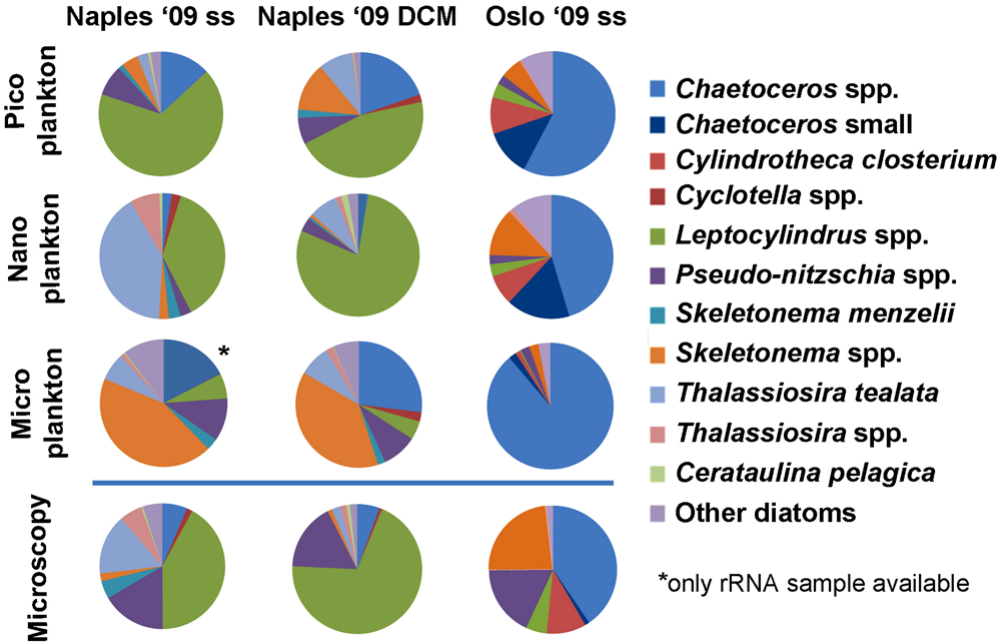
Species distribution in the three size-fractions and from light microscopy (non-fractionated) samples from Naples subsurface (ss) and DCM waters (October 2009) and Oslo subsurface waters (September 2009). rDNA and rRNA results were merged. Data from Oslo ’09 are from Dittami *et al*.^58^. The main taxa detected through metabarcoding matched those seen by light microscopy of the corresponding samples. The relative abundance of taxa from cell counts was generally more similar to that detected by sequences in the nanoplanktonic size fraction.

The large majority of the diatom genera to which reads were assigned include nano- and microplanktonic species (Fig. 2). Species of both these size classes were distributed in all three size fractions, although the picoplanktonic ones had generally lower numbers of diatom reads. For three sample sets with high diatom abundance (Naples subsurface and Naples DCM collected in October 2009 and Oslo subsurface collected in September 2009), taxa distribution in the distinct size-fractionated samples was analysed in greater detail and compared with light microscopy counts from the corresponding non-fractionated samples (Fig. 3). For each sampling point, the three size fractions were generally dominated by the same genera and even the same species in several cases. Smaller species (e.g. small *Chaetoceros* in the Oslo sample) were at times more abundant in the nanoplankton size-fraction, but in other cases (e.g. *Skeletonema menzelii* and *Thalassiosira tealata* in the Naples samples) they were abundant in the microplankton fraction as well. Overall, the main taxa detected in the metabarcoding dataset matched those seen in light microscopy of the corresponding samples, and the relative abundance of species in the nanoplankton size-fraction was closest to that determined from light microscopy results.

### Comparative analyses

**Figure 4:**
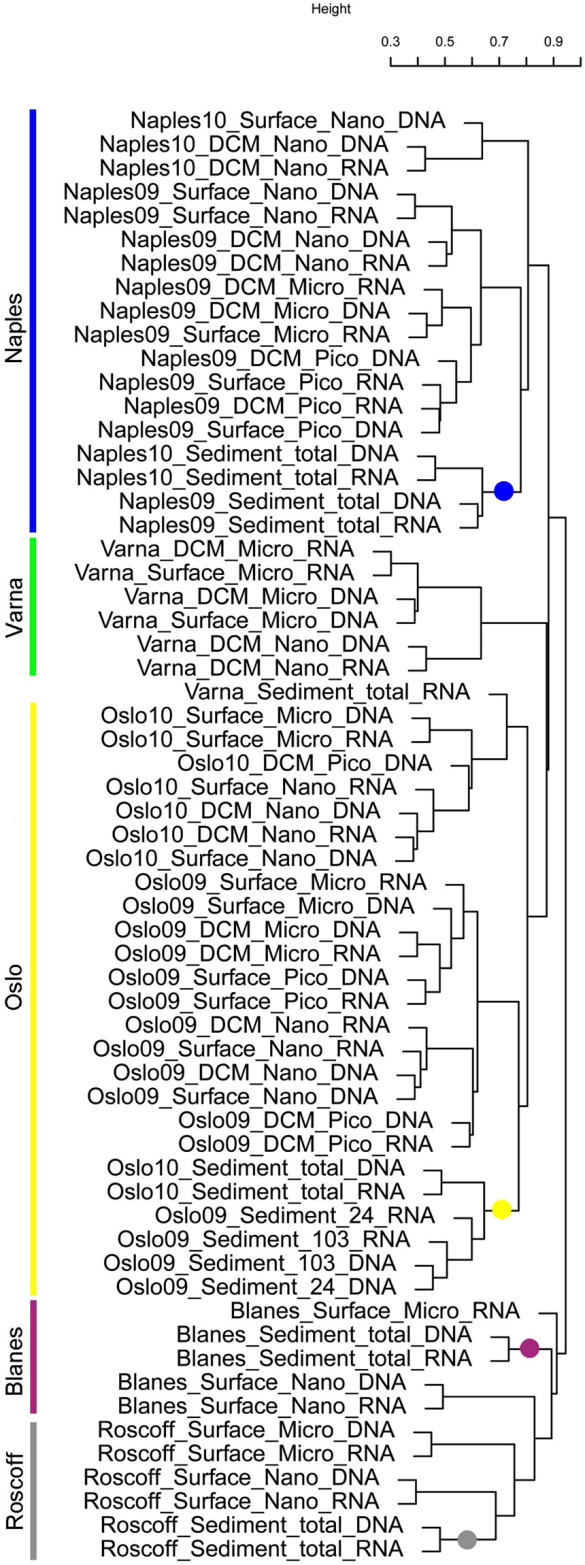
Cluster analysis of 61 diatom read samples based on a Bray-Curtis dissimilarity matrix of OTUs. The main clusters are related to the geographic origin of samples, as highlighted by coloured bars. The coloured dots indicate samples from sediments, which always clustered with the planktonic ones of the corresponding site. Blanes (Mediterranean) communities clustered with those from Roscoff (Atlantic Ocean) while Varna (Black Sea) communities clustered with those from Oslo (Skagerrak).

In agreement with the results of taxonomic analyses, clear differences among sites were highlighted by an OTU 99%-based cluster analysis of all samples consisting of more than 100 reads each (Supplementary Table S1). All samples from individual sites clustered together, including template pairs (rRNA and rDNA), size fractions of subsurface and DCM samples, and sediment samples. For the two sites (Naples and Oslo) that were sampled twice in different seasons, the water column samples grouped by dates whereas the sediment samples collected on different dates clustered together. Geographic patterns were apparently less obvious. Samples from Varna (Black Sea) and Blanes did not form a Mediterranean group with the samples from Naples but clustered with Skagerrak (Oslo) and Atlantic samples (Roscoff), respectively (Fig. 4).

rRNA and rDNA results for the same samples were generally paired in the cluster analyses. However, the two templates showed marked differences. The 89,158 rRNA reads grouped into almost three times more OTUs at 99% than the 53,878 rDNA reads (3,984 and 1,405 OTUs, respectively). In the analysis with normalized read numbers (Supplementary Table S5), the two templates shared only 17% of OTUs, but these included the large majority of the reads (95.1%). The high diversity of the rRNA datasets involved a high number of exclusive OTUs (69% of the total OTUs, 4.2% of the total read number), compared with 14% (0.7% of the total read number) of OTUs only found in rDNA datasets (Supplementary Table S5). In a comparison based on the taxonomic assignment, more than one third of the named species or genera (122) were only found in the rRNA dataset, while only 9 were exclusively found in the rDNA one. Yet the number of taxa shared between the two templates was much higher (220 taxa, 64%) than the shared OTU number and they included 99.5% of the total number of reads.

Considerable differences were also found in diatom diversity between the water column and the sediments. The two habitats shared 10% of OTUs, which included the largest part of the reads, with OTUs exclusively found in the water column or sediments being 52% and 36% of the normalized OTU number, respectively (Supplementary Table S5). About half of the 340 taxa identified based on the read assignment (173 taxa) were shared between the two habitats and they included 98% of the reads. Regarding the remaining taxa, one quarter were exclusively detected in the water column and one quarter in the sediments. The taxa exclusively detected in the water column included truly planktonic species such as several *Pseudo-nitzschia* species, *Fragilariopsis*, *Rhizosolenia*, *Proboscia*, *Leptocylindrus aporus*, *L*. *convexus*, *Tenuicylindrus belgicus*, *Cyclotella*, and various *Chaetoceros* species (mostly subgenus Hyalochaetae) but also about 30 benthic taxa mostly represented by a few reads. Diatoms only detected in sediment samples were various benthic pennate taxa, generally represented by a few reads most often only assigned at the genus level. There were also some planktonic species only recorded in the sediments, and they mainly belonged to spore-forming species.

**Figure 5:**
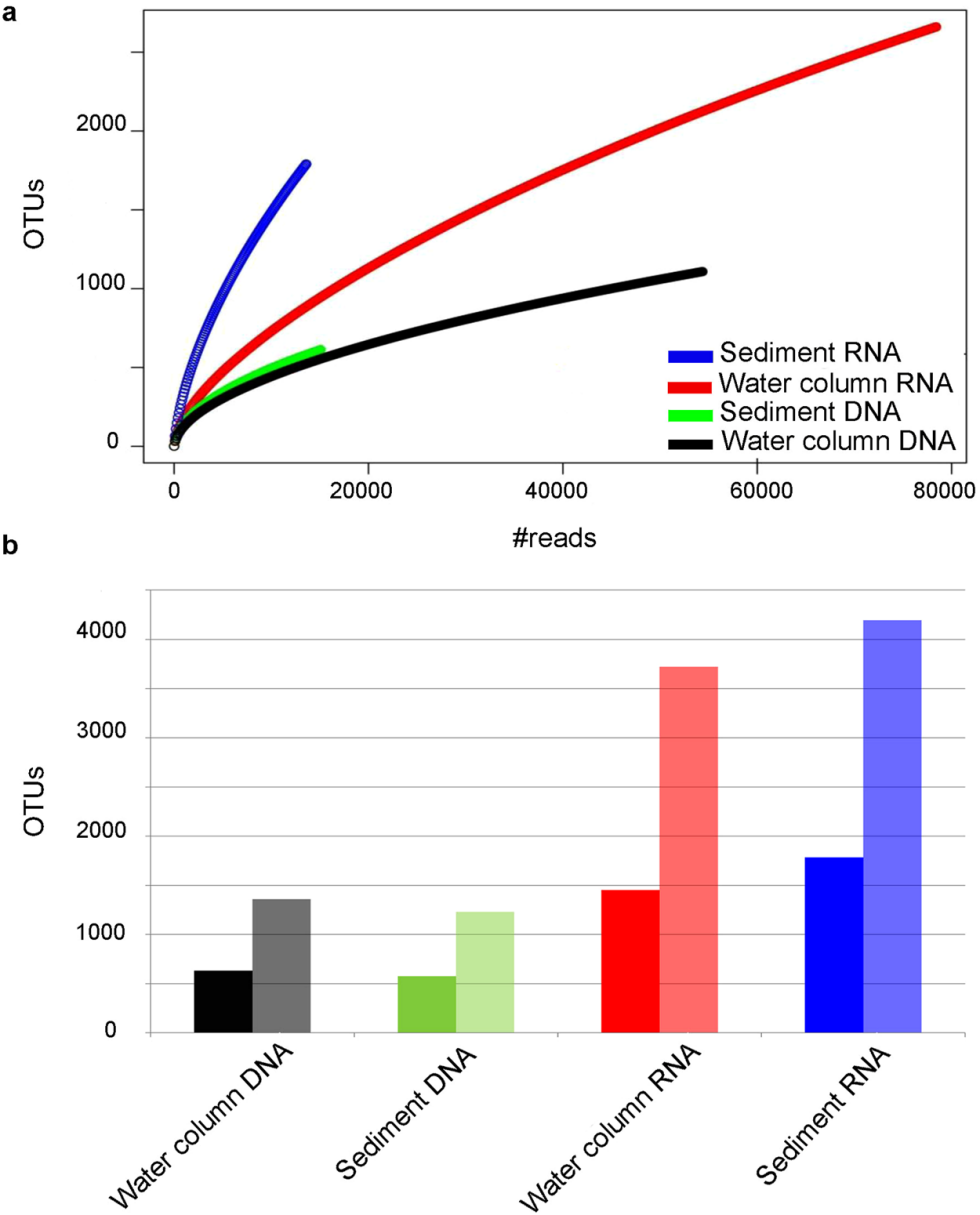
Alpha diversity in the four groups of pooled diatom read samples from water column and sediment rDNA and rRNA. (**a**) Rarefaction curve of observed OTUs showing that rRNA is always more diverse than rDNA (blue and red *vs.* green and black) and sediment rRNA more diverse than water column rRNA (blue *vs.* red). (**b**) Observed (dark colour) and estimated (from chao index, light colour) OTU richness in the water column and sediments and for the two templates based on a normalized read number, confirming higher diversity values in rRNA than in rDNA.

Rarefaction curves for samples pooled into four groups (rDNA and rRNA from water column and sediments, Supplementary Table S2) showed rRNA to be more diverse than rDNA in both water column and sediment samples. Moreover, in rDNA the diversity pattern was similar between water column and sediment samples, whereas in rRNA the pooled sediment samples exhibited a higher diversity than the ones of the water column (Fig. 5a,b). The Shannon index confirmed these results showing higher diversity values in rRNA (4.82 and 5.41 in water column and sediment samples, respectively) than rDNA (3.63 and 3.53 for water column and sediment samples, respectively) (Supplementary Table S6). The overall higher diversity in rRNA reflected the results from several individual sites, especially those with high diatom read numbers. At several sites (e.g., Blanes and Naples) diatom diversity was higher in the sediments than in the water column, but this pattern was less evident or even inverted in some cases, such as the one of sediments collected at 103 m in Oslo 2009, which were less diverse than the corresponding water column samples (Supplementary Fig. S2 and Supplementary Table S3).

## Discussion

This high-throughput sequencing investigation, based on the V4 region of 18S, on plankton and sediment from six European sites provides a first overview of diatom distribution across different coastal areas, revealing shared features but also peculiarities with respect to the whole protistan community.

The diatom species detected in large number of reads at European sites in this study are already known to be important in European coastal waters based on classical diatom surveys^19–22^. This is not surprising, considering the good correspondence found for the relative abundance of diatom taxa between metabarcoding and light microscopy data^12,23^. The dominance of *Leptocylindrus* in the whole dataset mainly reflects a bloom of species of this genus, which reached the density of up to 9 × 10^5^ cells l^−1^ during the autumn sampling in the Gulf of Naples. In fact the autumn 2009 samples from Naples and Oslo (dominated by *Chaetoceros*) were the richest in reads, covering about 75% of the total diatom dataset. Genera such as *Chaetoceros*, *Thalassiosira* and *Skeletonema*, which were the other most abundant taxa across coastal sites, are also found among the most abundant in offshore areas^12^. Interestingly, in the present coastal dataset *Chaetoceros* consists mostly of the Hyalochaetae subgenus (i.e., *Chaetoceros* with thin setae lacking plastids), whereas the Phaeoceros group (species with the plastids also in the thick setae) is much more represented in the prevalently offshore Tara Ocean dataset^12^. The dominance of Hyalochaetae versus Phaeoceros in coastal areas is consistent with previous reports^24^ and with the distribution of the two subgenera depicted in the OBIS maps (Ocean Biogeographic Information System (OBIS). 2012. *Chaetoceros*. http://www.iobis.org/mapper/?taxon_id=611649). It is also in line with the life strategy of most Hyalochaetae which, differently from Phaeoceros, form benthic resting stages that in deep waters would be less likely to return to the surface again.

Several species to which metabarcodes were assigned represent new findings for the Skagerrak and Gulf of Naples waters, where diatoms have been intensively investigated over the years with classical methods. These new findings should be interpreted cautiously, considering that the 18S rDNA sequence may vary by just a few base pairs among some congeneric species^25,26^. Nevertheless, at times not even electron microscopy suffices to identify species that differ by subtle ultrastructural features or are identical (cryptic species). In this respect, metabarcoding nicely complements classic studies while stimulating deeper taxonomic analyses to achieve a more accurate description of the actual diversity and distribution of diatom species in the natural environment.

Planktonic diatoms widely dominate in the water column but also in sediments, where they indicate the preponderance of resting stages and the possible contribution of cells recently settled from the water column. However, benthic taxa mostly contribute to the high species richness. The bulk of diatom biodiversity actually resides within benthic and tychopelagic species, which evolved and diversified in a broad variety of habitats while only a fraction of them acquired an almost exclusively planktonic life-style^7,27^. This is evident in the smaller number of exclusively planktonic genera compared with the large number of genera of benthic diatoms^28^. The higher diversity of diatom communities in the sediments, however, seems not to be the rule in this group of mostly autotrophic protists, for which lower irradiance at greater depths can limit photosynthesis, as it probably was the case for the sediment communities of the deep Oslo and Varna samples.

A peculiarity of diatoms with respect to other protists is the close relationships among assemblages across the different layers of the water column and the sediments at individual sites. These results markedly diverge from the pattern emerging from the whole protistan community^16,17^, which showed that sediment samples from different sites were closer among themselves than to the corresponding water column samples, pointing at a benthic protistan community well distinct from the planktonic one. Indeed, diatoms are one of the few cases among protists for which a high overlap exists between the sediment and water-column communities^17^, a condition stemming from the resting stage strategies, which are widespread in centric diatom genera (*Chaetoceros*, *Skeletonema* and *Thalassiosira*) dominating coastal assemblages^23,29^. In addition, the tychopelagic habit of many benthic species (mainly raphid pennates) also contributes to the similarity of diatom assemblages between the two habitats. Indeed, many prevalently benthic species are often found in the water column, where they are passively resuspended or actively move through buoyancy changes, thus increasing their dispersal potential. Interestingly, the typically planktonic pennate *Pseudo-nitzschia* spp., which are not known to produce resting stages, are virtually absent from the sediments, indicating that some species have an exclusively planktonic life-style even if they are hardly seen in the plankton most of the year. The relative rarity of *Leptocylindrus* in sediment samples compared to surface waters may also look unexpected since some species in this genus do form resting spores^25,30^. However, the most abundant species in the sampling period (and in the whole dataset) was *L*. *aporus*^10^, which does not form resting stages. These results highlight the need for regular comparison of benthic and planktonic samples in combination with sediment traps in order to assess the benthic-pelagic coupling along the seasons and the fate of the resting stages raining down on the sediments.

Altogether, the close relationship of all samples within individual sites, including different size fractions, habitats and even sampling dates, highlights a marked individuality of each site in terms of diatom communities. This situation is consistent with that reported for freshwater diatom communities^31^, in which a strong prevalence of microevolutionary processes generates distinct regional species pools, similar to what happens for larger organisms. We believe that the signal of geographic individuality is even more evident in the molecular signatures and would probably go undetected in case we used the taxonomic information, as many species are shared among sites, but they may consist of different locally adapted populations^32^. Thus, a series of filters related to dispersal, historical, evolutionary and ecological processes appears to shape local communities with site-specific characteristics.

On the other hand, the large-scale geographic patterns emerging from diatom community distribution are not obvious from our results. Particularly within the Mediterranean region, the clustering of samples from the Spanish coasts (Blanes) with those from the Eastern Atlantic (Roscoff) rather than with those of the closest Tyrrhenian waters (Naples), also revealed by the whole protistan communities^16^, probably reflects the stronger influence of surface Atlantic waters along the western Mediterranean coasts especially in winter. Interestingly, the Black Sea community (Varna) is completely different from the one from Naples, and rather clusters with that from Oslo, with which Varna shares similar environmental conditions, i.e. low salinity in surface waters, and much lower temperature in winter compared to the Mediterranean Sea. The relationship between Varna and Oslo communities is also reinforced by the presence in the Black Sea of relict cold water species, a memory of its geological history already noticed in the case of the genus *Leptocylindrus*^10^. Clearly, a realistic appraisal of large-scale geographic patterns and the understanding of their driving factors require more extensive surveys including different seasons.

In this study it has been possible to assign almost all reads to known genera and 91% of them to individual species. Those are much higher numbers compared to other assignments regarding the whole protistan community^33,34^ and metabarcoding studies of diatoms in offshore waters sampled during the Tara Oceans expedition^12^ or in benthic freshwater environments^35^. The higher percentage of assignment in the present study reflects the advanced knowledge of diatoms in coastal waters, especially in European seas. There, diatoms have been relatively well investigated and a large number of species have been brought into culture, identified and barcoded^25,36–38^. An exception seems to be constituted by benthic diatoms, in which many ribotypes are quite different (>2% sequence divergence) from the reference sequences of genetically characterised species, particularly for *Amphora*, *Cymbella*, *Fallacia*, *Gyrosigma* and *Pleurosigma*, all representatives of the raphid pennate lineage. This situation may derive from the low coverage of reference sequences for the numerous taxa described in this lineage based on morphology, but also from the existence of still undescribed taxa. In either case, this points at many remaining knowledge gaps for diversity even in this group of well-known organisms. Overall, diatoms largely support the view based on the whole protist assemblages that most of the unknown marine diversity is in the benthic realm^17^.

The high level of assignment of ribotypes to known taxa allows for some considerations on the use of OTUs as a proxy for the number of species. In the whole diatom dataset, the number of OTUs is much higher than that of distinct taxa to which reads were assigned, the two numbers drawing nearer only at an OTU cutoff of 93% sequence similarity (Supplementary Table S7**)**, which is a far too low threshold for diversity analysis. Even assuming a possible underestimation of the species number based on taxonomic assignment, and taking into account diversity concealed in the unassigned ribotypes, the lack of match between these two diversity units remains remarkable. It should also be considered that the variability of the chosen marker tag, V4, is not homogenous across diatom genera and species. For example, some species such as *Leptocylindrus danicus* may include several different OTUs even at 97% similarity^10^, whereas in other cases (e.g., in the genera *Skeletonema* and *Pseudo-nitzschia*) some species may be indistinguishable based on V4. Therefore, even within a single taxonomic group such as diatoms, it is not possible to find a conversion factor allowing a straightforward comparison between OTUs and species numbers.

From the technical point of view, the peculiar distribution of diatoms in different size-fractionated samples, with a scarce contribution to the picoplankton, but with the same species encountered in all three size fractions, can be explained in several ways. First, as already discussed for *Leptocylindrus*^10^, diatom cells or colonies can have elongated or complex shapes. Therefore the same specimen can pass through, or be trapped by the same plankton net depending on its angle of approach. Second, diatom cell size diminishes markedly over the course of its vegetative life cycle. Since a population can exhibit a range of cell sizes^39^, smaller specimens pass through the net whereas bigger ones remain trapped. In centric diatoms, a third possible source of high variance in size derives from the formation of flagellated gametes, which are markedly smaller than the vegetative cells. Therefore, a broad distribution of reads across size fractions is expected for individual diatom species compared to the majority of other protistan species, which are often globular in shape and rather homogeneous in size, and thus preferentially found in single size fractions. As a matter of fact, diatoms found in the picoplanktonic fraction are not those characterised by very small cell size and, especially for pennate diatoms, which do not form tiny flagellate gametes, their finding is probably caused by their rupture during filtration. Similarly, small-sized species found in the microplanktonic fraction are possibly captured because of net clogging, or because they were trapped in fecal pellets. From our results, it appears that the nanoplankton size fraction most closely reflects the actual relative contribution of diatom taxa in the plankton as deduced from light microscopy cell counts. However, both cell-size distribution and clogging rates may vary across different samples and lead to different outcomes of size fractionation which, in any case, is not informative and hence not advisable in the study of diatoms.

The analysis of the BioMarKs diatom dataset provides some new insights in the characterization of microbial assemblages with rDNA and rRNA. The two templates are rarely used in parallel in eukaryotic surveys, although interest in comparing the use of either of the two molecules is growing^40,41^. In our study, the pairing of the two templates for each sample confirms the picture that emerged from the whole protistan community^16^: the main compositional patterns of diatom communities are consistently reflected by the two templates and the community structures obtained with either template in different sites are comparable. However, while both templates are able to describe in the same way the 95% of the dataset, remarkable differences are evident in the rare component of diversity, which is captured more effectively by rRNA than by rDNA. In particular, high fractions of OTUs are exclusively found in the rRNA whereas a few are exclusively found in the rDNA. The higher diversity exclusive for the rRNA (in terms of OTUs or taxa) is in agreement with previous bacterial and protistan studies that suggest the rRNA-based approach be more effective in the description of the rare but active component of diversity, which is often lost with the rDNA^1,42–45^. This is however not true for other studies on bacteria^46^ or metazoa from marine sediments^47,48^. Nevertheless, these contrasting results from different surveys are expected and congruent with the well-known complex relationship between rRNA concentrations and differences in life strategies, life histories or metabolic activity among the different taxa^49^, calling for dedicated studies that elucidate variations in rRNA-rDNA relationships at intra-group, intra-specific and also intra-individual level.

The difference in the performance between the two templates particularly affects the comparison between the two habitats explored in this study, whereby rRNA describes higher diversity than rDNA especially in sediment communities. This result is unexpected, since sediments harbour a large quantity of extracellular rDNA accumulated over the seasons^50^ which, along with dormant resting stages, should result in a higher diversity for rDNA than rRNA, the latter deemed to better represent the active, viable community. On the other hand, both extracellular and dead cell rDNA might be so abundant in sediments, e.g. as a consequence of a past bloom, as to be overrepresented and mask the actual diversity of the sample, which is instead fully shown in the rRNA samples.

In conclusion, diatom HTS metabarcoding shows a high potential in shedding light on several questions on diversity, distribution, physiological activity and life history aspects, which are not fully understood even in this group of protists deemed to be well known. In addition, taking into account the considerable amount of time and expertise required for identification, diatom metabarcoding can effectively represent a valid complementary or alternative approach to classical methods for water quality assessment^51–53^, and certainly highly adequate to be translated into automated *in situ* technologies. Nonetheless, an effort is still required towards taxonomic studies that improve the reference datasets for the annotation of environmental tag sequences. The use of more variable marker sequences, e.g. LSU or even ITS, might allow for a sounder attribution of closely related species in metabarcoding analyses. However, in the case of diatoms, 18S fragments can already resolve taxa at the species level in many genera^10,38^, which makes more detailed analyses of existing HTS-metabarcoding datasets worthwhile and able to produce interesting insights into the ecology and distribution of diatoms in the world’s seas.

## Methods

Marine samples were collected within the course of the BioMarKs project (http://biomarks.eu/) at 6 coastal sites along the European coast near Blanes (BBMO station, Balearic Sea, Spain), Gijón (Gulf of Biscay, Spain), Naples (LTER-MC station, Gulf of Naples, Tyrrhenian Sea, Italy), Oslo (Skagerrak, Norway), Roscoff (SOMLIT station, Western English Channel, France) and Varna (Black Sea, Bulgaria) (Table 1). In Naples and Oslo the sampling was conducted on two dates. For details on sampling protocols, nucleic acid extraction, 454 pyrosequencing of the V4 18S rDNA region and data pre-processing see Massana *et al*.^16^. Briefly, seawater samples were collected along with environmental parameters at two depths of the water column, named Subsurface and DCM in the original dataset and corresponding to ca. 1 m depth and to a depth between 10 and 40 m, not always corresponding to an actual deep chlorophyll maximum (DCM), respectively. Seawater samples were collected on filters for the evaluation of three different size fractions. The 3–20 μm (nanoplankton) and 0.8–3 μm (picoplankton) fractions were obtained from ca. 20 litres of seawater, first prefiltered through a metallic sieve of 20 μm and then sequentially filtered with a peristaltic pump through 3 μm and 0.8 μm polycarbonate (PC) membranes (142 mm diameter). The 20–2,000 μm (micro-mesoplankton) samples were collected using a 20 μm-mesh plankton net, then pre-filtered through a 2,000 μm sieve and afterwards collected onto polycarbonate membrane PC membranes (47 mm diameter, 12 μm pore size). Samples were also collected from the sediments at depths between 20 and 103 m without size fractionation. The complete V4 BioMarKs sequence dataset is available at the European Nucleotide Archive under the study accession number PRJEB9133.

**Table 1:** Summary of sampling sites, diatom read numbers and environmental parameters.

Sampling				Reads		Environmental parameters		
Site	Coordinates	Date	Depth (m)	rDNA	rRNA	Temperature (°C)	Salinity	Chl a (μg L^−1^)
Blanes	41°40′N, 02°48′E	09/02/2010	Subsurface (1)	440	3294	12.5	37.6	0.7
			Sediment (20)	126	2554	12.6	37.8	—
Gijón	43°40′N, 5°35′W	14/09/2010	Subsurface (1)	76	45	20.2	35.7	0.6
Naples	40°48′N, 14°15′E	13/10/2009	Subsurface (1)	8985	29667	22.8	37.7	1.7
			DCM (26)	7300	18388	19.2	37.9	1.5
			Sediment (78)	5484	5524	14.6	37.9	—
		14/05/2010	Subsurface (1)	276	91	19.2	37.2	1.1
			DCM (34)	305	326	15.5	37.7	1
			Sediment (78)	1587	879	14	37.9	—
Oslo	59°16′N, 10°43′E	22/09/2009	Subsurface (1)	7023	8046	15.5	25.2	2.5
			DCM (20)	2212	6179	16.1	29.2	1.1
			Sediment (103)	1904	1074	8.2	35	—
			Sediment (24)	2057	1451	16.2	29.7	—
		22/06/2010	Subsurface (1)	3105	888	15	21.5	1.1
			DCM (10)	5433	4056	11.9	29.5	1.9
			Sediment (103)	3596	975	6	35	—
Roscoff	48°46′N, 3°57′W	20/04/2010	Subsurface (1)	1746	3815	9.9	34.9	0.2
			Sediment (60)	264	795	9.9	34.9	—
Varna	43°10′N, 28°50′E	27/05/2010	Subsurface (3)	1292	358	18	16.5	5.2
			DCM (40)	665	443	8.7	17.9	6.1
			Sediment (386)	3	311	8.8	21.8	—

From the V4 total cleaned BioMarKs protist datasets for two templates, rDNA and rRNA, the dissolved rDNA samples, duplicates and samples from anoxic sediment from Varna were removed and the metabarcodes (or unique ribotypes) assigned to Bacillariophyta were extracted. The initial taxonomic assignation of reads was confirmed and refined performing a local blastn against a custom version of the PR^2^ database (10.6084/m9.figshare.5913181) containing also unpublished diatom sequences. A total of 491 reads, corresponding to 0.24% of the total Bacillariophyceae dataset, had similarity ≤90% and query coverage ≤70% to reference sequences and therefore were removed. Unique ribotypes were hence assigned to species’ reference sequences at similarity level between 98 and 100%, and to genera for similarity levels lower than 98%.

For phytoplankton identification and abundance assessment in light microscopy, samples were collected with Niskin bottles and immediately fixed with neutralized formaldehyde at a final concentration of 0.8%. Subsamples of 5–10 ml were allowed to settle in sedimentation chambers, and cells were counted in transects or the whole chamber bottom with an inverted light microscope (Zeiss Axiovert or Nikon Eclipse TE300) at ×400 magnification using the Utermöhl method^54^.

The taxonomic overview of diatom distribution across the sites was performed at genus level and based on all V4 reads merged by individual sites (Supplementary Table S3), pooling together the different size fractions, depth and templates.

The distribution of genera in the water column and sediments and across the sites were visualised through a treemap graph generated using Treemap package in R. Treemap presents data in hierarchical structure which uses rectangles the area size of which is proportional to the quantities.

To explore alpha and beta-diversity, we generated an OTUs table at 99% using the vsearch distance-based greedy clustering algorithm (method = dgc) through mothur^55,56^. Additional thresholds were also explored (Supplementary Table S7**)**. The alpha diversity was explored at different levels. For templates and habitats we defined four groups of reads (Water Column DNA, Water Column RNA, Sediment DNA and Sediment RNA, Supplementary Table S2), whereby different depths (subsurface and DCM) and size fractions (meso, nano and pico) were grouped and cumulated for each site. Rarefaction curves of observed OTUs in the four groups were built. Moreover, we calculated observed OTU (sobs), projected OTU numbers (chao) and the Shannon index, after a random subsampling to the number of reads present in the community with the lowest amount of reads (Sediment RNA = 13,653, Supplementary Table S2). The comparison of the four groups of reads was also performed at site level except for Gijón, which contained only 121 reads. In the latter analysis the normalization was performed at site level (Supplementary Table S3).

The number of OTUs shared between total rDNA samples and total rRNA samples and between total water column samples and total benthos samples was obtained after normalization to the number of reads present in the group with least reads  (Total DNA = 53,879 in the templates comparison and Total sediment = 28,584 in the habitats comparison, Supplementary Table S3).

For the cluster analysis, the samples with less than 100 reads were removed and the remaining 61 samples (Supplementary Table S1) were subsampled to 415 reads using the tool ‘rrarefy’ in the VEGAN R package^57^, and then log transformed, to diminish the effect of the most abundant OTUs. A distance matrix was computed with the Bray-Curtis index, and a dendrogram was constructed using the ‘average’ method.

## Electronic supplementary material

Supplementary information

Supplementary Table S4
