# Supplementary material for: Diatom diversity through HTS-metabarcoding in coastal European seas
Source: Sci Rep. 2018 Dec 24;8:18059. doi: 10.1038/s41598-018-36345-9 (PMC6305388; doi:10.1038/s41598-018-36345-9)
Supplement: Supplementary file 1 — Supplementary information [file 41598_2018_36345_MOESM1_ESM.pdf]

Diatom diversity through HTS-metabarcoding in coastal European seas

Roberta Piredda, Jean-Michel Claverie, Johan Decelle, Colomban de Vargas, Micah Dunthorn, Bente Edvardsen, Wenche Eikrem, Dominik Forster, Wiebe H. C. F. Kooistra, Ramiro Logares, Ramon Massana, Marina Montresor, Fabrice Not, Hiroyuki Ogata, Jan Pawlowski, Sarah Romac, Diana Sarno, Thorsten Stoeck and Adriana Zingone

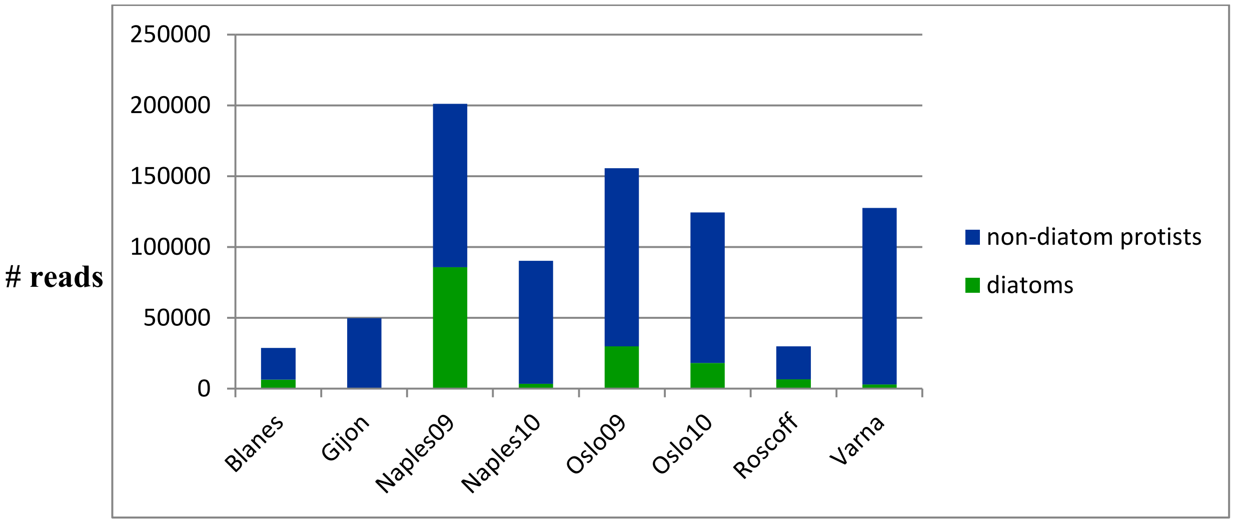

Supplementary Figure S1. Abundance of diatom and other protist reads at the six sampling sites.

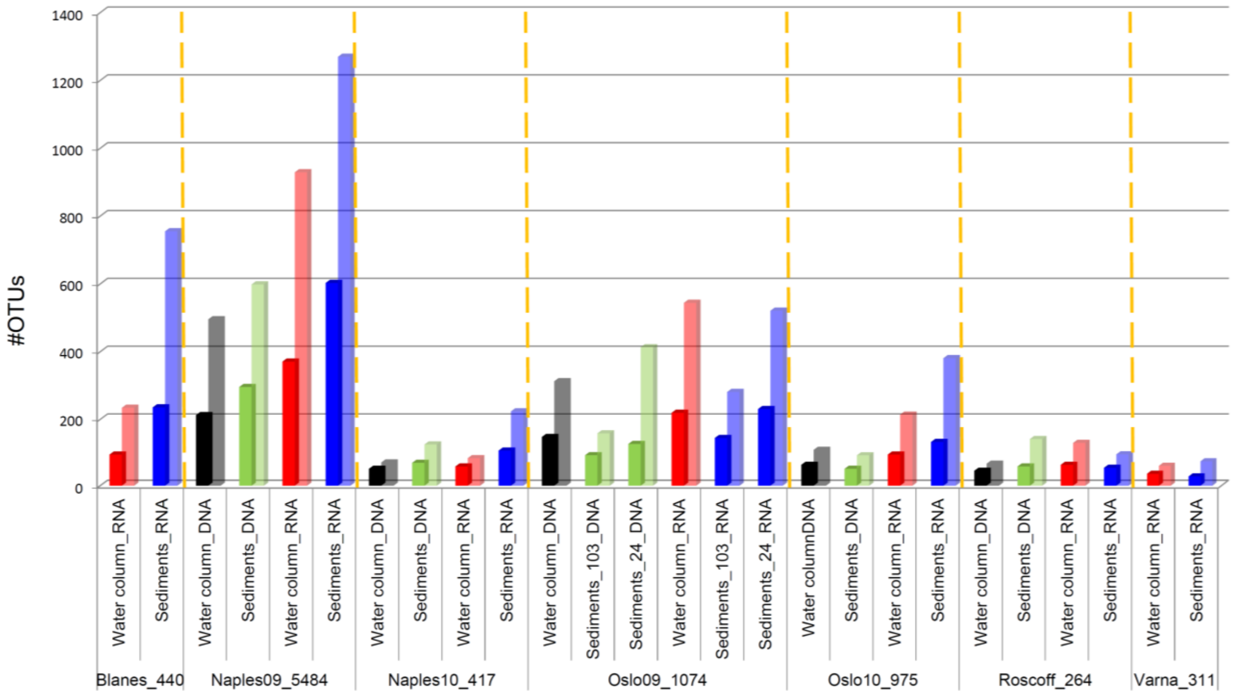

Supplementary Figure S2. Observed (dark colour) and estimated diversity (from chao index, light colour) in the four groups of samples pooled by template and by habitat at each sampling site. The number after each site name corresponds to the number of sequences normalised among the four groups at each site.

Supplementary Table S1. List of BioMarKS diatom read samples used in this study. Colour codes in column B correspond to those merged and used in Fig. 1a to show the distribution of diatom genera in the water column (green nuances) and sediments (purple nuances). Colour codes in column D correspond to those used in Fig. 1b and Fig. 4 to indicate different sampling sites. Bold in column E indicates samples used for the cluster analysis shown in Fig. 4.

Samples	Samples codes	#reads
Blanes_Subsurface_Micro_DNA	S0044	19
**Blanes_Subsurface_Micro_RNA**	S0155	360
**Blanes_Subsurface_Nano_DNA**	S0076	387
**Blanes_Subsurface_Nano_RNA**	S0066	2860
Blanes_Subsurface_Pico_DNA	S0077	34
Blanes_Subsurface_Pico_RNA	S0067	74
**Blanes_Sediment_total_DNA**	S0078	126
**Blanes_Sediment_total_RNA**	S0068	2553
Gijon_Subsurface_Nano_DNA	S0160	70
Gijon_Subsurface_Nano_RNA	S0158	39
Gijon_Subsurface_Pico_DNA	S0159	6
Gijon_Subsurface_Pico_RNA	S0157	6
Naples09_Subsurface_Micro_DNA	S0125	33
**Naples09_Subsurface_Micro_RNA**	S0135	3101
**Naples09_Subsurface_Nano_DNA**	S0047	8169
**Naples09_Subsurface_Nano_RNA**	S0166	24777
**Naples09_Subsurface_Pico_DNA**	S0169	783
**Naples09_Subsurface_Pico_RNA**	S0163	1789
**Naples09_DCM_Micro_DNA**	S0170	453
**Naples09_DCM_Micro_RNA**	S0164	1996
**Naples09_DCM_Nano_DNA**	S0171	6561
**Naples09_DCM_Nano_RNA**	S0165	15698
**Naples09_DCM_Pico_DNA**	S0168	286
**Naples09_DCM_Pico_RNA**	S0162	694
**Naples09_Sediment_total_DNA**	S0008	5484
**Naples09_Sediment_total_RNA**	S0005	5524
Naples10_Subsurface_Micro_DNA	S0083	72
Naples10_Subsurface_Micro_RNA	S0073	18
**Naples10_Subsurface_Nano_DNA**	S0084	150
Naples10_Subsurface_Nano_RNA	S0074	58
Naples10_Subsurface_Pico_DNA	S0095	54
Naples10_Subsurface_Pico_RNA	S0085	15
Naples10_DCM_Micro_DNA	S0096	76
Naples10_DCM_Micro_RNA	S0086	63
**Naples10_DCM_Nano_DNA**	S0097	223

**Naples10_DCM_Nano_RNA**	S0087	245
Naples10_DCM_Pico_DNA	S0098	6
Naples10_DCM_Pico_RNA	S0088	18
**Naples10_Sediment_total_DNA**	S0099	1587
**Naples10_Sediment_total_RNA**	S0089	879
**Oslo09_Subsurface_Micro_DNA**	S0075	2159
**Oslo09_Subsurface_Micro_RNA**	S0065	2924
**Oslo09_Subsurface_Nano_DNA**	S0142	3832
**Oslo09_Subsurface_Nano_RNA**	S0132	3530
**Oslo09_Subsurface_Pico_DNA**	S0141	1032
**Oslo09_Subsurface_Pico_RNA**	S0131	1592
**Oslo09_DCM_Micro_DNA**	S0138	636
**Oslo09_DCM_Micro_RNA**	S0128	3100
**Oslo09_DCM_Nano_DNA**	S0136	1413
**Oslo09_DCM_Nano_RNA**	S0126	2797
**Oslo09_DCM_Pico_DNA**	S0137	163
**Oslo09_DCM_Pico_RNA**	S0127	282
**Oslo09_Sediment_103_DNA**	S0139	1903
**Oslo09_Sediment_103_RNA**	S0129	1074
**Oslo09_Sediment_24_DNA**	S0140	2057
**Oslo09_Sediment_24_RNA**	S0130	1451
**Oslo10_Subsurface_Micro_DNA**	S0118	122
**Oslo10_Subsurface_Micro_RNA**	S0108	453
**Oslo10_Subsurface_Nano_DNA**	S0117	2950
**Oslo10_Subsurface_Nano_RNA**	S0107	415
Oslo10_Subsurface_Pico_DNA	S0116	33
Oslo10_Subsurface_Pico_RNA	S0106	20
**Oslo10_DCM_Nano_DNA**	S0115	5283
**Oslo10_DCM_Nano_RNA**	S0105	3999
**Oslo10_DCM_Pico_DNA**	S0143	150
Oslo10_DCM_Pico_RNA	S0133	57
**Oslo10_Sediment_total_DNA**	S0144	3596
**Oslo10_Sediment_total_RNA**	S0134	975
**Roscoff_Subsurface_Micro_DNA**	S0079	285
**Roscoff_Subsurface_Micro_RNA**	S0069	756
**Roscoff_Subsurface_Nano_DNA**	S0080	1406
**Roscoff_Subsurface_Nano_RNA**	S0070	2976
Roscoff_Subsurface_Pico_DNA	S0081	55
Roscoff_Subsurface_Pico_RNA	S0071	83
**Roscoff_Sediment_total_DNA**	S0082	264
**Roscoff_Sediment_total_RNA**	S0072	795
**Varna_Subsurface_Micro_DNA**	S0100	1238
**Varna_Subsurface_Micro_RNA**	S0090	339

Varna_Subsurface_Nano_DNA	S0101	42
Varna_Subsurface_Nano_RNA	S0091	19
Varna_Subsurface_Pico_DNA	S0102	12
**Varna_DCM_Micro_DNA**	S0103	473
**Varna_DCM_Micro_RNA**	S0093	301
**Varna_DCM_Nano_DNA**	S0104	170
**Varna_DCM_Nano_RNA**	S0094	122
Varna_DCM_Pico_DNA	S0122	22
Varna_DCM_Pico_RNA	S0112	20
Varna_Sediment_total_DNA	S0123	3
**Varna_Sediment_total_RNA**	S0113	311

Supplementary Table S2. List of samples grouped by habitat and template with their corresponding number of reads, as used in Figs. 5.

Water Column rDNA

grouped samples	#reads
Naples09_DCM_Micro_DNA	453
Naples09_DCM_Nano_DNA	6561
Naples09_DCM_Pico_DNA	286
Naples10_DCM_Micro_DNA	76
Naples10_DCM_Nano_DNA	223
Naples10_DCM_Pico_DNA	6
Oslo09_DCM_Micro_DNA	636
Oslo09_DCM_Nano_DNA	1413
Oslo09_DCM_Pico_DNA	163
Oslo10_DCM_Nano_DNA	5283
Oslo10_DCM_Pico_DNA	150
Varna_DCM_Micro_DNA	473
Varna_DCM_Nano_DNA	170
Varna_DCM_Pico_DNA	22
Blanes_Subsurface_Micro_DNA	19
Blanes_Subsurface_Nano_DNA	387
Blanes_Subsurface_Pico_DNA	34
Gijon_Subsurface_Nano_DNA	70
Gijon_Subsurface_Pico_DNA	6
Naples09_Subsurface_Micro_DNA	33
Naples09_Subsurface_Nano_DNA	8169
Naples09_Subsurface_Pico_DNA	783
Naples10_Subsurface_Micro_DNA	72
Naples10_Subsurface_Nano_DNA	150
Naples10_Subsurface_Pico_DNA	54
Oslo09_Subsurface_Micro_DNA	2159
Oslo09_Subsurface_Nano_DNA	3832
Oslo09_Subsurface_Pico_DNA	1032
Oslo10_Subsurface_Micro_DNA	122
Oslo10_Subsurface_Nano_DNA	2950
Oslo10_Subsurface_Pico_DNA	33
Roscoff_Subsurface_Micro_DNA	285
Roscoff_Subsurface_Nano_DNA	1406
Roscoff_Subsurface_Pico_DNA	55
Varna_Subsurface_Micro_DNA	1238
Varna_Subsurface_Nano_DNA	42
Varna_Subsurface_Pico_DNA	12
**TOTAL**	**38858**

Water Column rRNA

grouped samples	#reads
Naples09_DCM_Micro_RNA	1996
Naples09_DCM_Nano_RNA	15698
Naples09_DCM_Pico_RNA	694
Naples10_DCM_Micro_RNA	63
Naples10_DCM_Nano_RNA	245
Naples10_DCM_Pico_RNA	18
Oslo09_DCM_Micro_RNA	3100
Oslo09_DCM_Nano_RNA	2797
Oslo09_DCM_Pico_RNA	282
Oslo10_DCM_Nano_RNA	3999
Oslo10_DCM_Pico_RNA	57
Varna_DCM_Micro_RNA	301
Varna_DCM_Nano_RNA	122
Varna_DCM_Pico_RNA	20
Blanes_Subsurface_Micro_RNA	360
Blanes_Subsurface_Nano_RNA	2860
Blanes_Subsurface_Pico_RNA	74
Gijon_Subsurface_Nano_RNA	39
Gijon_Subsurface_Pico_RNA	6
Naples09_Subsurface_Micro_RNA	3101
Naples09_Subsurface_Nano_RNA	24777
Naples09_Subsurface_Pico_RNA	1789
Naples10_Subsurface_Micro_RNA	18
Naples10_Subsurface_Nano_RNA	58
Naples10_Subsurface_Pico_RNA	15
Oslo09_Subsurface_Micro_RNA	2924
Oslo09_Subsurface_Nano_RNA	3530
Oslo09_Subsurface_Pico_RNA	1592
Oslo10_Subsurface_Micro_RNA	453
Oslo10_Subsurface_Nano_RNA	415
Oslo10_Subsurface_Pico_RNA	20
Roscoff_Subsurface_Micro_RNA	756
Roscoff_Subsurface_Nano_RNA	2976
Roscoff_Subsurface_Pico_RNA	83
Varna_Subsurface_Micro_RNA	339
Varna_Subsurface_Nano_RNA	19
**TOTAL**	**75596**

Sediment rDNA

grouped samples	#reads
Blanes_Sediment_total_DNA	126
Naples09_Sediment_total_DNA	5484
Naples10_Sediment_total_DNA	1587
Oslo09_Sediment_103_DNA	1903
Oslo09_Sediment_24_DNA	2057
Oslo10_Sediment_total_DNA	3596
Roscoff_Sediment_total_DNA	264
Varna_Sediment_total_DNA	3
**TOTAL**	**15020**

Sediment rRNA

grouped samples	#reads
Blanes_Sediment_total_RNA	2553
Naples09_Sediment_total_RNA	5524
Naples10_Sediment_total_RNA	879
Oslo09_Sediment_103_RNA	1074
Oslo09_Sediment_24_RNA	1451
Oslo10_Sediment_total_RNA	975
Roscoff_Sediment_total_RNA	795
Varna_Sediment_total_RNA	311
**TOTAL**	**13562**

Supplementary Table S3. Summary of sample grouping and number of sequences available for analyses represented in the Suppl. Fig. S2.

	Water column		Total water column	Sediments		Deep sediments		Total sediments
**Site**	**rDNA**	rRNA		**rDNA**	rRNA	**rDNA**	rRNA	
**Blanes**	440	3294	3734	126	2553			2679
**Gijon**	76	45	121					
**Naples_09**	16285	48055	64340	5484	5524			11008
**Naples_10**	581	417	998	1587	879			2466
**Oslo_09**	9235	14225	23460	2056	1451	1904	1074	6485
**Oslo_10**	8538	4944	13482	3596	975			4571
**Roscoff**	1746	3815	5561	264	795			1059
**Varna**	1957	801	2758	3	311			314
TOTAL	**38858**	**75596**	**114454**	**13116**	**12488**	**1904**	**1074**	**28582**
			**Total rDNA**	**53878**				
			**Total rRNA**	**89158**				

(Supplementary Table 4, see excel file)

Supplementary Table S5. Percent of OTUs shared or exclusive among subsets (reads in brackets). Total Template is based on merged habitats, while Total Habitats is based on merged templates. rRNA showed the highest number of exclusive OTUs and sequences in both the water column and the sediments.

Total Template		
	rDNA	rRNA
percent shared OTUs (% seqs)	17% (95%)	
percent exclusive OTUs (% seqs)	14% (0.7%)	69% (4.2%)
**Water Column**		
	rDNA	rRNA
percent shared OTUs (% seqs)	35% (92%)	
percent exclusive OTUs (% seqs)	12% (1.2%)	52% (5.8%)
**Sediments**		
	rDNA	rRNA
**percent shared OTUs (% seqs)**	14% (87%)	
**percent exclusive OTUs (% seqs)**	10% (1.3%)	60% (10%)
**Total Habitat**		
	Column	Sediment
percent shared OTUs (% seqs)	10% (80%)	
percent exclusive OTUs (% seqs)	52% (12%)	36% (8.6%)

Supplementary Table S6. Summary of normalized alpha diversity in the four groups of samples merged by template and habitat (see Supplementary Table S2).

	#seqs	sobs	chao	shannon
**Water Column_rDNA**	13563	632	1359	3.63
**Sediment_rDNA**	13563	576	1230	3.53
**Water Column_rRNA**	13563	1451	3722	4.82
**Sediment_rRNA**	13563	1784	4192	5.41

Supplementary Table S7. Number of OTUs generated at different thresholds of similarity. The number of OTUs at the threshold of 93% represented the best match with the number of taxonomic assignation (341 taxa).

**Clustering threshold**	**99%**	**98%**	**97%**	**96%**	**95%**	**94%**	**93%**	**92%**
**#OTUs**	4587	1740	1102	784	580	435	343	275

Supplementary Dataset 1. *Skeletonema tropicum* and S. *pseudocostatum* V4 sequences.

>DQ396517.1_S._tropicum_strain_CCMP_2070

AGCTCCAATAGCGTATATTAAAGTTGTTGCAGTTAAAAAGCTCGTAGTTGGATTTCTGGC

AGGAGTGACCGACCACACACACCGTGTGTGAGTTGTGTCATTCTGGCCATCCTTGGTGAG

ATCCTATTTGGCATTAAGTTGTCGGGTAGGGGATAACCATCGTTTACTGTGAAAAAATTA

GAGTGTTTAAAGCAGGCTTATGCCGTTGAATATATTAGCATGGAATAATAAGATAGGACT

TTGAGTCTATTTTGTTGGTTTGCGAGTCAAAGTAATGATTAATAGGGACAGTTGGGGGTA

TTCGTATTTCATTGTCAGAGGTGAAATTCTTGGATTTCTGAAAGACGAACTACTGCGAAA

GCATTTACCAAGGATGTTTTCA

>DQ396516.1_S._tropicum_strain_B210

AGCTCCAATAGCGTATATTAAAGTTGTTGCAGTTAAAAAGCTCGTAGTTGGATTTCTGGC

AGGAGTGACCGACCACACACACCGTGTGTGAGTTGTGTCATTCTGGCCATCCTTGGTGAG

ATCCTATTTGGCATTAAGTTGTCGGGTAGGGGATAACCATCGTTTACTGTGAAAAAATTA

GAGTGTTTAAAGCAGGCTTATGCCGTTGAATATATTAGCATGGAATAATAAGATAGGACT

TTGAGTCTATTTTGTTGGTTTGCGAGTCAAAGTAATGATTAATAGGGACAGTTGGGGGTA

TTCGTATTTCATTGTCAGAGGTGAAATTCTTGGATTTCTGAAAGACGAACTACTGCGAAA

GCATTTACCAAGGATGTTTTCA

>DQ396515.1_S._tropicum_strain_B205

AGCTCCAATAGCGTATATTAAAGTTGTTGCAGTTAAAAAGCTCGTAGTTGGATTTCTGGC

AGGAGTGACCGACCACACACACCGTGTGTGAGTTGTGTCATTCTGGCCATCCTTGGTGAG

ATCCTATTTGGCATTAAGTTGTCGGGTAGGGGATAACCATCGTTTACTGTGAAAAAATTA

GAGTGTTTAAAGCAGGCTTATGCCGTTGAATATATTAGCATGGAATAATAAGATAGGACT

TTGAGTCTATTTTGTTGGTTTGCGAGTCAAAGTAATGATTAATAGGGACAGTTGGGGGTA

TTCGTATTTCATTGTCAGAGGTGAAATTCTTGGATTTCTGAAAGACGAACTACTGCGAAA

GCATTTACCAAGGATGTTTTCA

>X85393.1_S._pseudocostatum_strain_CCAP_1077/6

AGCTCCAATAGCGTATATTAAAGTTGTTGCAGTTAAAAAGCTCGTAGTTGGATTTCTGGC

AGGAGTGACCGACCACACACACCGTGTGTGAGTTGTGTCATTCTGGCCATCCTTGGTGAG

ATCCTATTTGGCATTAAGTTGTCGGGTAGGGGATAACCATCGTTTACTGTGAAAAAATTA

GAGTGTTTAAAGCAGGCTTATGCCGTTGAATATATTAGCATGGAATAATAAGATAGGACT

TTGAGTCTATTTTGTTGGTTTGCGAGTCAAAGTAATGATTAATAGGGACAGTTGGGGGTA

TTCGTATTTCATTGTCAGAGGTGAAATTCTTGGATTTCTGAAAGACGAACTACTGCGAAA

GCATTTACCAAGGATGTTTTCA

>X85394.1_S._pseudocostatum_strain_CCAP_1077/7

AGCTCCAATAGCGTATATTAAAGTTGTTGCAGTTAAAAAGCTCGTAGTTGGATTTCTGGC

AGGAGTGACCGACCACACACACCGTGTGTGAGTTGTGTCATTCTGGCCATCCTTGGTGAG

ATCCTATTTGGCATTAAGTTGTCGGGTAGGGGATAACCATCGTTTACTGTGAAAAAATTA

GAGTGTTTAAAGCAGGCTTATGCCGTTGAATATATTAGCATGGAATAATAAGATAGGACT

TTGAGTCTATTTTGTTGGTTTGCGAGTCAAAGTAATGATTAATAGGGACAGTTGGGGGTA

TTCGTATTTCATTGTCAGAGGTGAAATTCTTGGATTTCTGAAAGACGAACTACTGCGAAA

GCATTTACCAAGGATGTTTTCA

>AJ632208.1_S._pseudocostatum_strain_SZN-B78

AGCTCCAATAGCGTATATTAAAGTTGTTGCAGTTAAAAAGCTCGTAGTTGGATTTCTGGC

AGGAGTGACCGACCACACACACCGTGTGTGAGTTGTGTCATTCTGGCCATCCTTGGTGAG

ATCCTATTTGGCATTAAGTTGTCGGGTAGGGGATAACCATCGTTTACTGTGAAAAAATTA

GAGTGTTTAAAGCAGGCTTATGCCGTTGAATATATTAGCATGGAATAATAAGATAGGACT

TTGAGTCTATTTTGTTGGTTTGCGAGTCAAAGTAATGATTAATAGGGACAGTTGGGGGTA

TTCGTATTTCATTGTCAGAGGTGAAATTCTTGGATTTCTGAAAGACGAACTACTGCGAAA

GCATTTACCAAGGATGTTTTCA

>AB948148.1_S._pseudocostatum_strain_FMR120

AGCTCCAATAGCGTATATTAAAGTTGTTGCAGTTAAAAAGCTCGTAGTTGGATTTCTGGC

AGGAGTGACCGACCACACACACCGTGTGTGAGTTGTGTCATTCTGGCCATCCTTGGTGAG

ATCCTATTTGGCATTAAGTTGTCGGGTAGGGGATAACCATCGTTTACTGTGAAAAAATTA

GAGTGTTTAAAGCAGGCTTATGCCGTTGAATATATTAGCATGGAATAATAAGATAGGACT

TTGAGTCTATTTTGTTGGTTTGCGAGTCAAAGTAATGATTAATAGGGACAGTTGGGGGTA

TTCGTATTTCATTGTCAGAGGTGAAATTCTTGGATTTCTGAAAGACGAACTACTGCGAAA

GCATTTACCAAGGATGTTTTCA

>AJ632206.1_S._pseudocostatum_strain_CCAP1077-7

AGCTCCAATAGCGTATATTAAAGTTGTTGCAGTTAAAAAGCTCGTAGTTGGATTTCTGGC

AGGAGTGACCGACCACACACACCGTGTGTGAGTTGTGTCATTCTGGCCATCCTTGGTGAG

ATCCTATTTGGCATTAAGTTGTCGGGTAGGGGATAACCATCGTTTACTGTGAAAAAATTA

GAGTGTTTAAAGCAGGCTTATGCCGTTGAATATATTAGCATGGAATAATAAGATAGGACT

TTGAGTCTATTTTGTTGGTTTGCGAGTCAAAGTAATGATTAATAGGGACAGTTGGGGGTA

TTCGTATTTCATTGTCAGAGGTGAAATTCTTGGATTTCTGAAAGACGAACTACTGCGAAA

GCATTTACCAAGGATGTTTTCA

Supplementary Dataset 2. *Pseudo-nitzschia australis* and *P. multiseries* V4 sequences.

>JN599166.1_P_australis

AGCTCCAATAGCGTATATTAAAGTTGTTGCAGTTAAAAAGCTCGTAGTTGGATTTGTGGT

GTGTCCAGTCGGCCTTTGCTCTTTGAGTGATTGCGCTGTACTGGTCTGCCATGTTTGGGT

GGAATCTGTGTGGCATTAAGTTGTCGTGCAGGGGATGCCCATCGTTTACTGTGAAAAAAT

TAGAGTGTTCAAAGCAGGCTTATGCCGTTGAATATATTAGCATGGAATAATGATATAGGA

CCTTGGTACTATTTTGTTGGTTTGCGCACTAAGGTAATGATTAAGAGGGACAGTTGGGGG

TATTTGTATTCCATTGTCAGAGGTGAAATTCTTGGATTTT-TGGAAGACAAACTACTGCG

AAAGCATTTACCAAGGATGTTTTCA

>GU373964.1_P_multiseries

AGCTCCAATAGCGTATATTAAAGTTGTTGCAGTTAAAAAGCTCGTAGTTGGATTTGTGGT

GTGTCCAGTCGGCCTTTGCTCTTTGAGTGATTGCGCTGTACTGGTCTGCCATGTTTGGGT

GGAATCTGTGTGGCATTAAGTTGTCGTGCAGGGGATGCCCATCGTTTACTGTGAAAAAAT

TAGAGTGTTCAAAGCAGGCTTATGCCGTTGAATATATTAGCATGGAATAATGATATAGGA

CCTTGGTACTATTTTGTTGGTTTGCGCACTAAGGTAATGATTAAGAGGGACAGTTGGGGG

TATTTGTATTCCATTGTCAGAGGTGAAATTCTTGGATTTT-TGGAAGACAAACTACTGCG

AAAGCATTTACCAAGGATGTTTTCA

>U18241.1_P_multiseries

AGCTCCAATAGCGTATATTAAAGTTGTTGCAGTTAAAAAGCTCGTAGTTGGATTTGTGGT

GTGTCCAGTCGGCCTTTGCTCTTTGAGTGATTGCGCTGTACTGGTCTGCCATGTTTGGGT

GGAATCTGTGTGGCATTAAGTTGTCGTGCAGGGGATGCCCATCGTTTACTGTGAAAAAAT

TAGAGTGTTCAAAGCAGGCTTATGCCGTTGAATATATTAGCATGGAATAATGATATAGGA

CCTTGGTACTATTTTGTTGGTTTGCGCACTAAGGTAATGATTAAGAGGGACAGTTGGGGG

TATTTGTATTCCATTGTCAGAGGTGAAATTCTTGGATTTT-TGGAAGACAAACTACTGCG

AAAGCATTTACCAAGGATGTTTTCA

>AM235381.1_P_multiseries_strain_TKA_2-28

AGCTCCAATAGCGTATATTAAAGTTGTTGCAGTTAAAAAGCTCGTAGTTGGATTTGTGGT

GTGTCCAGTCGGCCTTTGCTCTTTGAGTGATTGCGCTGTACTGGTCTGCCATGTTTGGGT

GGAATCTGTGTGGCATTAAGTTGTCGTGCAGGGGATGCCCATCGTTTACTGTGAAAAAAT

TAGAGTGTTCAAAGCAGGCTTATGCCGTTGAATATATTAGCATGGAATAATGATATAGGA

CCTTGGTACTATTTTGTTGGTTTGCGCACTAAGGTAATGATTAAGAGGGACAGTTGGGGG

TATTTGTATTCCATTGTCAGAGGTGAAATTCTTGGATTTT-TGGAAGACAAACTACTGCG

AAAGCATTTACCAAGGATGTTTTCA

>AY221947.1_P_multiseries

AGCTCCAATAGCGTATATTAAAGTTGTTGCAGTTAAAAAGCTCGTAGTTGGATTTGTGGT

GTGTCCAGTCGGCCTTTGCTCTTTGAGTGATTGCGCTGTACTGGTCTGCCATGTTTGGGT

GGAATCTGTGTGGCATTAAGTTGTCGTGCAGGGGATGCCCATCGTTTACTGTGAAAAAAT

TAGAGTGTTCAAAGCAGGCTTATGCCGTTGAATATATTAGCATGGAATAATGATATAGGA

CCTTGGTACTATTTTGTTGGTTTGCGCACTAAGGTAATGATTAAGAGGGACAGTTGGGGG

TATTTGTATTCCATTGTCAGAGGTGAAATTCTTGGATTTT-TGGAAGACAAACTACTGCG

AAAGCATTTACCAAGGATGTTTTCA

>U18241.1_P_multiseries

AGCTCCAATAGCGTATATTAAAGTTGTTGCAGTTAAAAAGCTCGTAGTTGGATTTGTGGT

GTGTCCAGTCGGCCTTTGCTCTTTGAGTGATTGCGCTGTACTGGTCTGCCATGTTTGGGT

GGAATCTGTGTGGCATTAAGTTGTCGTGCAGGGGATGCCCATCGTTTACTGTGAAAAAAT

TAGAGTGTTCAAAGCAGGCTTATGCCGTTGAATATATTAGCATGGAATAATGATATAGGA

CCTTGGTACTATTTTGTTGGTTTGCGCACTAAGGTAATGATTAAGAGGGACAGTTGGGGG

TATTTGTATTCCATTGTCAGAGGTGAAATTCTTGGATTTT-TGGAAGACAAACTACTGCG

AAAGCATTTACCAAGGATGTTTTCA

>GU373961.1_P_australis

AGCTCCAATAGCGTATATTAAAGTTGTTGCAGTTAAAAAGCTCGTAGTTGGATTTGTGGT

GTGTCCAGTCGGCCTTTGCTCTTTGAGTGATTGCGCTGTACTGGTCTGCCATGTTTGGGT

GGAATCTGTGTGGCATTAAGTTGTCGTGCAGGGGATGCCCATCGTTTACTGTGAAAAAAT

TAGAGTGTTCAAAGCAGGCTTATGCCGTTGAATATATTAGCATGGAATAATGATATAGGA

CCTTGGTACTATTTTGTTGGTTTGCGCACTAAGGTAATGATTAAGAGGGACAGTTGGGGG

TATTTGTATTCCATTGTCAGAGGTGAAATTCTTGGGTTTT-TGGAAGACAAACTACTGCG

AAAGCATTTACCAAGGATGTTTTCA

>AM235382.1_P_multiseries_strain_POMX

AGCTCCAATAGCGTATATTAAAGTTGTTGCAGTTAAAAAGCTCGTAGTTGGATTCGTGGT

GTGTCCAGTCGGCCTTTGCTCTTTGAGTGATTGCGCTGTACTGGTCTGCCATGTTTGGGT

GGAATCTGTGTGGCATTAAGTTGTCGTGCAGGGGATGCCCATCGTTTACTGTGAAAAAAT

TAGAGTGTTCAAAGCAGGCTTATGCCGTTGAATATATTAGCATGGAATAATGATATAGGA

CCTTGGTACTATTTTGTTGGTTTGCGCACTAAGGTAATGATTAAGAGGGACAGTTGGGGG

TATTTGTATTCCATTGTCAGAGGTGAAATTCTTGGATTTT-TGGAAGACAAACTACTGCG

AAAGCATTTACCAAGGATGTTTTCA

>AM235380.1_P_multiseries_strain_Nparl

AGCTCCAATAGCGTATATTAAAGTTGTTGCAGTTAAAAAGCTCGTAGTTGGATTCGTGGT

GTGTCCAGTCGGCCTTTGCTCTTTGAGTGATTGCGCTGTACTGGTCTGCCATGTTTGGGT

GGAATCTGTGTGGCATTAAGTTGTCGTGCAGGGGATGCCCATCGTTTACTGTGAAAAAAT

TAGAGTGTTCAAAGCAGGCTTATGCCGTTGAATATATTAGCATGGAATAATGATATAGGA

CCTTGGTACTATTTTGTTGGTTTGCGCACTAAGGTAATGATTAAGAGGGACAGTTGGGGG

TATTTGTATTCCATTGTCAGAGGTGAAATTCTTGGATTTT-TGGAAGACAAACTACTGCG

AAAGCATTTACCAAGGATGTTTTCA
